# The relationship of online pre-recorded neurology mini-lectures to medical student assessment: a pilot study

**DOI:** 10.1186/s12909-023-04185-5

**Published:** 2023-04-07

**Authors:** Hani TS Benamer, Adrian G Stanley

**Affiliations:** Department of Clinical Sciences, College of Medicine, Mohammed Bin Rashid University of Medicine and Health Sciences, United Arab Emirates, Building 14, PO Box 505055, Dubai, UAE

**Keywords:** Undergraduate Medical Education, Lecture, Medical students, Online learning, Nervous System Diseases

## Abstract

**Introduction:**

eLearning has become an essential part of medical education. However, there is a lack of published research on student engagement with online pre-recorded mini-lectures and its relation to assessment. The aim of this pilot study is to explore the relationship between newly introduced neurology pre-recorded mini-lectures and undergraduate medical students engagement and assessment. This may encourage the wider use of mini-lectures in undergraduate medical curricula.

**Methods:**

The engagement of medical students with 48 online pre-recorded neurology mini-lectures was assessed through a Learning Management System. To measure engagement, data was stratified according to the number of watched/downloaded mini-lectures. A point system was used (out of 5): − 1 point = watching/downloading 0–10 mini-lectures, 2 points = watching/downloading 11–20 mini-lectures, 3 points = watching/downloading 21–30 mini-lectures, 4 points = watching/downloading 31–40 mini-lectures and, 5 points = watching/downloading 41–48 mini-lectures. The students’ engagement was correlated with their neurology assessments [Objective Structured Clinical Examination (OSCE), and knowledge-based assessment 10 Multiple Choice Questions (MCQs) and one 10-mark Short Answer Question, (SAQ)], internal medicine grade and annual grade point average (GPA) using the Pearson correlation coefficient.

**Results:**

The mean engagement of 34, Year 5, medical students is 3.9/5. There is a significant positive correlation between engagement and internal medicine grade (*r* = 0.35, p = 0.044). There is a moderate correlation between engagement and neurology OSCE (*r* = 0.23), annual Year 5 GPA (*r* = 0.23), neurology knowledge-based score (*r* = 0.22) and composite neurology knowledge/OSCE (r = 0.27). The knowledge-based assessment included SAQ and MCQs: there was a moderate correlation with SAQ (*r* = 0.30), but a weak negative correlation with the MCQs (*r* =-0.11). Sub-groups analysis comparing the top- and low- or non- engaging students made these weaker correlations stronger.

**Conclusion:**

This pilot study indicates a high rate of engagement with an online pre-recorded mini-lectures resource and evidence of moderate correlation between engagement and assessment. Online pre-recorded mini-lectures should be used more in delivering the curriculum contents of the clinical clerkships. Further studies are needed to evaluate the relation and the impact of the mini-lectures on assessment.

## Introduction

There are eight medical schools in the United Arab Emirates [1]. The various medical programmes must be approved by the Ministry of Education and accredited by the Commission for Academic Accreditation [1]. All but one of the programmes are 5–6 years long, taking students from secondary schools [1]. The College of Medicine in Mohammed Bin Rashid University of Medicine and Health Sciences (MBRU), in Dubai, was inaugurated in 2016 and its first cohort graduated in June 2022. The MBRU offer a 6-year MBBS course which is divided into three phases. Phase 1 (Year 1) covers the fundamental concepts in science. Phase 2 (Years 2–3) includes systems (e.g., neurosciences) and integrated medicine courses. In phase 3, (Years 4–6), the students rotate through a series of clinical clerkships, the final year of which is based on an internship-style programme.

eLearning is an integral part of undergraduate medical education, enhanced since COVID-19. Dost et al. in a national cross-sectional survey of 2721 medical students, across 39 UK medical schools, reported significant difference between time spend on online material before and during the pandemic (7.35% versus 23.56%) [2]. This large study suggested that incorporation of online teaching methods could play a significant role in the future of medical education [2]. Most studies have shown a high rate of student engagement with online lectures [3, 4] and a positive correlation between better exam performance and online lectures engagement [5–7]. However one showed a low rate of engagement and an inverse association between engagement and final grades [8]. In their scoping review, Tang et al. [9] concluded that *“integration of online lectures into undergraduate medical education is well-received by students and appears to improve learning outcomes”*.

In our medical school, the concept of pre-recorded online mini-lectures (duration less than 20 min) was introduced in the two weeks neurology placement, which is part of 8-week internal medicine (IM) sub-specialties clerkship (acute medicine, haematology/oncology, and intensive care). The 48-neurology pre-recorded mini-lectures (Microsoft PowerPoint with voice-over) were introduced as a co-curricular e-learning resource, available through a Learning Management System (LMS) and in addition to other resources, e.g. the neurology chapter of Kumar and Clark’s Clinical Medicine [10]. The mini-lectures are user-friendly, can be viewed on demand and replayed as required. The students were advised (not mandated by summative assessment) to use this resource. The mean duration is 11.1 min (4.2 to 18.5); in total 531 min and covers all the core subjects of undergraduate clinical neurology.

There is very little literature, if any, relating to pre-recorded online mini-lectures and therefore, this paper aims to assess students’ engagement with this newly introduced resource and to measure the relation of these on student assessment performance. This study was undertaken during 2021-22 and thus following a ‘cultural’ shift in student approach to learning following the COVID-19.

## Methods

### Study design

Student engagement was assessed through the LMS, which can determine if an individual student watched/downloaded a mini-lecture. To measure engagement, data was stratified according to the number of watched/downloaded mini-lectures. A point system was used (out of 5): 1 point = watching/downloading 0–10 mini-lectures, 2 points = watching/downloading 11–20 mini-lectures, 3 points = watching/downloading 21–30 mini-lectures, 4 points = watching/downloading 31–40 mini-lectures and, 5 points = watching/downloading 41–48 mini-lectures. The data was collected after the Year 5 final exams.

The IM clerkship is 25% of Year 5 curriculum/assessment: the others are surgery (25%), ObGyn (25%), paediatrics (12.5%) and emergency medicine (12.5%). To pass each clerkship, the student must attain at least a C- grade (scheme: F/D/D+/C-/C/C+/B-/B/B+/A-/A). Student knowledge and skills are assessed at the end of year. Neurology contributes 25% of the total marks towards the IM grade: one Objective Structured Clinical Examination (OSCE), 10 Multiple Choice Questions (MCQs) and one 10-mark Short Answer Question (SAQ). The annual grade point average (GPA) is a weighted composite of all Year 5 assessments. The students’ engagement was correlated to their neurology assessments (OSCE, knowledge (MCQs and SAQ), a composite knowledge score, a composite knowledge/OSCE score, IM grade and annual GPA. Analysis of student subgroups was conducted: six ‘low-engaging’ students (watching/downloading 0–17 mini-lectures) or two ‘non-engaging’ students (watching/downloading 0–10) with the 17 ‘top-engaging’ students (watching/downloading 47–48).

### Statistical analysis

The engagement data was downloaded from the LMS in to an excel sheet. The point-system, described in the study design section was used to express the students’ engagement. Pearson correlation coefficient (r) was used to correlate student mini-lectures engagement with assessment type. The level of significant was set to *P*-values < 0.05. The statistical analyses were performed using SSPS version 28.0.0.0 (190).

### Ethics approval

The study was approved by the MBRU ethical committee (IRB-2022-156).

## Results

There were 34 Year 5 medical students (76% female).

### Mini-lecture engagement

The mean student engagement was 3.9/5; 2 students (6%) scored 1 point (watched/download 0–10 mini-lectures), 8 students (24%) scored 2 points (11–20 mini-lectures), no students scored 3 points (21–30 mini-lectures), 4 students (12%) scored 4 points (31–40 mini-lectures), and 20 students (58%) scored 5 points (41–48 mini-lectures). Seventeen (50%) students watched/downloaded 47–48 mini-lectures; all eight students watched 47 mini-lectures missed the *welcome* lecture. One student watched none. There was no correlation between the mini-lectures duration and student engagement (*r*=-0.06).

### Correlation with assessment scores

Mean neurology MCQs score was 86% (60-100%), SAQ was 73% (10-100%), and OSCE was 73% (50-96%). The composite knowledge/OSCE score was 77% (45-94%). Two students achieved the top A grade in IM: 3 = A-, 9 = B+, 5 = B, 6 = B-, 4 = C+, 1 = C, 3 = D+, and 1 = D. Mean annual GPA was 3.15 (2.04–3.85).

There is a mild-moderate positive correlation between student engagement and assessment performance. Significantly, there is a positive correlation between engagement and a better IM grade, but only a trend revealing a positive correlation against the neurology-specific assessment outcomes. This was mostly observed in the neurology knowledge-based assessments. There was discrepancy between the SAQ (moderate positive correlation) and MCQs (little correlation). A mild-moderate positive correlation was noted with the annual GPA. Subgroup analysis augmented the data trends observed overall with some comparisons reaching statistical significance. (Table 1).

**Table 1:** Correlation between assessment type and student engagement (mini-lectures number)

Assessment Type	All students (n = 34)		Low-engaging (n = 6) v. top-engaging students (n = 17)		Non-engaging (n = 2) v. top-engaging students (n = 17)	
	R-value	P- value	R-value	P-value	R-value	P- value
Neurology OSCE	0.23	0.195	0.34	0.117	0.59	0.008*
Neurology SAQ Question	0.3	0.089	0.36	0.094	0.48	0.061
Neurology MCQ Questions	− 0.11	0.538	− 0.14	0.54	0.02	0.948
All Neurology Questions	0.22	0.206	0.27	0.209	0.38	0.11
Neurology Questions/OSCE Score	0.27	0.118	0.34	0.111	0.49	0.032*
Year 5 IM Grade	0.35	0.044*	0.56	0.006*	0.67	0.002*
Annual GPA	0.23	0.187	0.42	0.047*	0.45	0.056

*P-value < 0.05

## Discussion

This study revealed high student engagement with watching/downloading a series of online pre-recorded neurology mini-lectures. There was a mean engagement rate of 3.9/5; 50% of students watching/downloading all the curricular content. This rate is similar to Berg’s study [11], in which 37% watched all the lectures and 5% watched none. There is evidence that student attention reaches a low point after 10–15 min in a 50-minute lecture [12, 13], so we postulate that the short mini-lecture duration (mean = 11 min) is an important contributory factor. In this study, mini-lecture duration had no effect on student engagement probably because all min-lectures are short (the longest = 18.5 min).

These mini-lectures were comprehensive, covering almost all topics in the clerkship’s learning outcomes. Students consider neurology to be a difficult subject (neurophobia) [14]. This might encourage student engagement with additional teaching, particularly if delivered by a faculty tutor. This behaviour may also reflect student assumption that assessment content will be drawn from the mini-lectures. However, six students were non-engaging or low-engagers. We assume that this minority prefer other resources (e.g., advisory textbooks; other texts or internet-accessible material), but it would be difficult to robustly capture student engagement with other material.

There was a significant positive correlation between mini-lecture engagement and the IM grade. There was a moderate, but non-significant, positive correlation between engagement and neurology assessments. Sub-group analysis (low-engaging/non-engaging v. top-engaging students) tended to reveal more significant data. Nevertheless, student performance is never solely based on a single resource as other factors are contributory, e.g., prior knowledge, engagement in the clinical setting and general academic ability.

The discrepancy between the SAQ and MCQs correlation with engagement is a surprising. This may relate to the question difficulty: mean MCQs score was 86% and thus likely to be less discriminatory compared to the mean SAQ score of 73%.

This study has limitations. Student number is low, which may limit the data’s statistical significance. Thus, we are planning the same analysis for the next cohort. The LMS does not allow a definitive confirmation of whether students who downloaded the mini-lectures have watched them: we have made that logical assumption. Finally, the study does not explore the reasons why some students poorly engage with the mini-lectures. Thus, we will include a short questionnaire addressing this issue for the next cohort.

In conclusion, this study is the first, to the best of our knowledge, to investigate medical student engagement with a pre-recorded online mini-lecture series accompanying a clinical clerkship and determine its relationship with assessment performance. This pilot study indicates a high engagement rate and a positive correlation between engagement and better assessment outcomes. Online pre-recorded mini-lectures should be used more in delivering the curriculum contents of clinical clerkships. Further studies are needed to evaluate the relation and the impact of the mini-lectures on students’ engagement and assessments.

## Data Availability

The datasets used and/or analysed during the current study available from the corresponding author on reasonable request. All methods were carried out in accordance with international accepted guidelines and regulations.

## References

[1] Alameri H, Hamdy H, Sims D (2021). Medical education in the United Arab Emirates: Challenges and opportunities. Med Teach.

[2] Dost S, Hossain A, Shehab M, Abdelwahed A, Al-Nusair L (2020). Perceptions of medical students towards online teaching during the COVID-19 pandemic: a national cross-sectional survey of 2721 UK medical students. BMJ Open.

[3] Pilarski PP, Alan Johnstone D, Pettepher CC, Osheroff N (2008). From music to macromolecules: using rich media/podcast lecture recordings to enhance the preclinical educational experience. Med Teach.

[4] Topale L (2016). The strategic use of lecture recordings to facilitate an active and self-directed learning approach. BMC Med Educ.

[5] Prakash SS, Muthuraman N, Anand R (2017). Short-duration podcasts as a supplementary learning tool: perceptions of medical students and impact on assessment performance. BMC Med Educ.

[6] White LJ, McGowan HW, McDonald AC (2019). The Effect of Content Delivery Style on Student performance in anatomy. Anat Sci Educ.

[7] Burnette K, Ramundo M, Stevenson M, Beeson MS (2009). Evaluation of a web-based asynchronous pediatric emergency medicine learning tool for residents and medical students. Acad Emerg Med Off J Soc Acad Emerg Med.

[8] McNulty JA, Hoyt A, Gruener G, Chandrasekhar A, Espiritu B, Price R (2009). An analysis of lecture video utilization in undergraduate medical education: associations with performance in the courses. BMC Med Educ.

[9] Tang B, Coret A, Qureshi A, Barron H, Ayala AP, Law M (2018). Online Lectures in Undergraduate Medical Education: scoping review. JMIR Med Educ.

[10] Feather A, Randall D, Waterhouse M. Kumar and Clark’s Clinical Medicine. 10th edition. Elsevier; 2020.

[11] Berg R, Brand A, Grant J, Kirk JS, Zimmerman T. Leveraging Recorded Mini-Lectures to Increase Student Learning. 2014;:2.

[12] McKeachie W, Svinicki M. McKeachie’s teaching tips. Cengage Learning; 2013.

[13] Wankat PC. The effective, efficient professor: teaching, scholarship, and service. Allyn and Bacon Boston; 2002.

[14] Jozefowicz RF (1994). Neurophobia: the fear of neurology among medical students. Arch Neurol.

